# The role of exposure in the treatment of anxiety in children and adolescents: protocol of a systematic review and meta-analysis

**DOI:** 10.1186/s13643-020-01337-2

**Published:** 2020-04-27

**Authors:** Kathrin Schopf, Cornelia Mohr, Michael W. Lippert, Katharina Sommer, Andrea Hans Meyer, Silvia Schneider

**Affiliations:** 1 Mental Health Research and Treatment Center, Faculty of Psychology, Ruhr University Bochum, Massenbergstrasse 9-13, 44787 Bochum, Germany; 2 Faculty of Psychology, University of Basel, Missionsstrasse 62A, 4055 Basel, Switzerland

**Keywords:** Anxiety disorder, Cognitive behavioral therapy, CBT, Exposure, Children, Adolescents

## Abstract

**Background:**

In children and adolescents, anxiety disorders (ADs) are among the most prevalent mental disorders. While there is a solid empirical foundation to support CBT as an evidence-based treatment for childhood ADs, the mechanisms underlying the efficacy of CBT are not well explored. Exposure is assumed to be vital to the efficacy of CBT in ADs, but empirical evidence (e.g., dismantling studies) showing that exposure is indeed a vital element of effective treatments is relatively scarce. The proposed meta-analysis aims to investigate the role of exposure in reducing symptoms of anxiety among children and adolescents.

**Methods:**

A systematic search of several electronic databases including PubMed/MEDLINE, PsycINFO, Psyndex plus, Web of Science, Scopus, and EMBASE will be conducted (from inception onwards). We will include randomized and non-randomized clinical trials examining exposure and anxiety among children and adolescents. If feasible, we will also include experimental, quasi-experimental, and observational studies. The primary outcome will be improvement in anxiety levels (recovery or change in anxiety rating scale) after exposure. Three reviewers will independently screen all citations, abstract data, and full-text articles. The methodological quality (or risk of bias) of individual studies will be appraised using an appropriate tool. If feasible, we will conduct mixed effects meta-analysis. Additional analyses will be conducted to explore the potential sources of heterogeneity (e.g., dose of exposure, age group, methodological quality).

**Discussion:**

This systematic review and meta-analysis will examine the role of exposure in reducing symptoms of anxiety among youth. The review will provide information on the working mechanisms underlying the efficacy of CBT. Our findings will be of interest to mental health professionals, researchers, and policy makers who wish to support children and adolescents with anxiety disorders by guiding well-informed treatment decisions.

**Systematic review registration:**

PROSPERO (CRD42019128667).

## Background

In children and adolescents, anxiety disorders (ADs) are among the most prevalent mental disorders, with point prevalence rates ranging between 6.5 and 10% [1, 2]. Meta-analyses have demonstrated associations between parental anxiety disorders and parenting behaviors on one hand and anxiety among youth on the other hand [3, 4]. A large number of clinical trials have investigated the efficacy of cognitive behavior therapy (CBT) for ADs in children and adolescents [5–9]. Several systematic reviews and meta-analyses have been conducted to compare CBT against control conditions or alternative treatments [10–18], which have consistently shown that CBT is an effective treatment for ADs among youth. Yet, while there is a solid empirical foundation to support CBT as an evidence-based treatment for childhood ADs, the mechanisms underlying the efficacy of CBT and the question of how CBT works for whom and under which conditions have been less thoroughly explored [16, 19]. In their systematic review, James and colleagues [13] concluded that it is time to move efficacy research away from the question *if* CBT works towards the question *how* CBT works.

As a central element of CBT, exposure is assumed to be vital to its efficacy [13, 20–23]. This assumption is supported by the finding that 91% of successful treatments for childhood ADs contain exposure [12]. Moreover, in a recent analysis of a subsample of the Child/Adolescent Anxiety Multimodal Treatment Study (CAMS), the therapist-reported quantity of exposure was found to be associated with independently rated treatment success. Results showed that more intense exposure was associated with more favorable treatment outcomes. Furthermore, it was found that treatment success could be predicted by the amount of time spent on more difficult (as opposed to mild or moderate) exposure tasks [24]. A meta-analysis, however, reported no advantage of CBT containing exposure compared to CBT without exposure (*d* = 0.15) [16]. In contrast, results showed that effect sizes of exposure-based treatments were comparable to effect sizes of other variants of CBT (cognitive restructuring, behavioral rehearsal, operant procedures, relaxation). It should be noted that this study collapsed anxiety and depressive disorders as well as children, adolescents, and adults into one group. Therefore, results may not be representative for children and adolescents with ADs. Another meta-analysis reported effect sizes of CBT for children and adolescents with ADs to be related neither to the total number of exposure sessions nor to the proportion of sessions involving exposure [25]. This study, however, used a rather narrow definition of ADs, including only social phobia, generalized anxiety disorder, and separation anxiety disorder. Therefore, findings may not be representative for all youth ADs. In sum, the contribution of exposure in the efficacy of CBT for ADs among youth, although theoretically well-defined [23], remains empirically unclear.

The proposed meta-analysis aims to investigate the questions whether exposure-based treatments are more effective than non-exposure treatments and if the dose of exposure (e.g., number, frequency/density, duration of exposure sessions) is associated with treatment efficacy among children and adolescents with ADs and high levels of anxiety. Treatment efficacy will be measured primarily as recovery from AD(s) or improvement in anxiety levels using common anxiety rating scales. In addition to clinical trials (randomized and non-randomized), we will also include experimental, quasi-experimental, and observational studies examining the relation between exposure and anxiety among youth. If exposure is indeed a vital treatment ingredient, then two assumptions should hold true when tested empirically. First, interventions containing exposure should be more effective than interventions without exposure. Second, the higher the quantity of exposure, the more effective the intervention should be. Next to the quantitative aspects of exposure (e.g., number of exposures with fear eliciting object(s), duration of exposure), qualitative aspects (e.g., variability in exposure tasks, use of multiple fear stimuli, use of multiple contexts) are assumed to influence its efficacy [21]. Based on this assumption, we also plan to investigate whether intervention efficacy varies as a function of qualitative differences in exposure tasks. As the planned meta-analysis is the first to investigate this question, we cannot predict which qualitative data will be available. Therefore, we plan to examine and summarize to which extent exposure-based interventions differ qualitatively. Depending on the availability of data, we will then examine if qualitative differences in exposure moderate intervention efficacy. Furthermore, we aim to examine whether the role of exposure in intervention efficacy is moderated by patient age. Positive outcomes of CBT for ADs have been reported in children as young as 4 years of age [5, 8, 26]. As cognitive interventions require a certain level of cognitive maturity, positive outcomes of CBT in young children may primarily reflect children’s response to behavioral rather than cognitive elements of CBT. Consequently, the role of exposure may be assumed to be greater in younger children.

## Methods

### Study registration

The present protocol has been registered within the PROSPERO database (registration number CRD42019128667) and is being reported in accordance with the reporting guidance provided in the Preferred Reporting Items for Systematic Reviews and Meta-Analyses Protocols (PRISMA-P) statement [27] (see checklist in Additional file 1).

### Eligibility criteria

In order to be included in this review, studies have to meet four inclusion criteria. First, we will include child and adolescent samples with an age range between 0 and 21 years. Second, participants will have to display either elevated symptoms of anxiety (including trait anxiety), as defined by the respective study, or a primary diagnosis of one or more of the following ADs: separation anxiety disorder, specific/simple phobia, social phobia/social anxiety disorder, generalized anxiety disorder/overanxious disorder, panic disorder, agoraphobia, selective/elective mutism. Inclusion will not be restricted by method of diagnosis, but we will code whether diagnoses were established using a standardized diagnostic interview. Third, studies will be included if they include at least one exposure-based intervention condition. For example, studies may compare exposure-based interventions against (a) a control condition without exposure (e.g., waitlist, treatment-as-usual, attention placebo), (b) another “bona fide” psychological treatment with or without exposure, (c) a pharmacological treatment, or (d) a combined psychological and pharmacological treatment with or without exposure. Bona fide treatments will be defined according to Tolin [16] and will be identified using criteria proposed by Wampold and colleagues [28] and Westen and colleagues [29]. We will code the degree to which each condition contains exposure. We will compare interventions with exposure to interventions without exposure and, if feasible, interventions with more exposure to interventions with less exposure. Fourth, regarding the study design, we will include randomized controlled trials (parallel-group or cluster-randomized) and non-randomized controlled trials. If feasible, we will also include experimental, quasi-experimental, and observational studies (observational studies are eligible, if they include a pre-post comparison). Exclusion criteria will pertain to the primary diagnosis of study participants. In accordance with the DSM-5 classification of ADs, studies investigating patients with post-traumatic stress disorder (PTSD) or obsessive-compulsive disorder (OCD) will be excluded if this is the primary diagnosis, but included if PTSD and/or OCD are diagnosed as comorbid disorders in addition to one or more ADs. Inclusion will not be restricted with regard to setting, outcomes, assessment points, publication form or status, year of publication, or language.

### Search strategy

Two search strategies will be employed to identify studies on exposure in childhood ADs. First, clinical trials will be identified through a systematic search of electronic databases (i.e., from inception onwards). The following databases will be searched (search platforms in parenthesis): PubMed (including MEDLINE; Ovid), PsycINFO (including PsycArticles; EBSCOhost), Psyndex plus (EBSCOhost), Web of Science (Thomson Reuters, Clarivate Analytics), Scopus (Elsevier), and Embase (Elsevier). We will conduct a search of titles, abstracts, keywords, and subject and/or thesaurus terms using four sets of key terms referring to [1] age of subjects, [2] diagnosis/symptoms of subjects, [3] intervention, and [4] study design (based on eligibility criteria). The search string was developed and piloted in PubMed and will be adapted to meet the specific requirements of each database. A draft search strategy for PubMed is provided in Additional file 2. No further limiters will be used. The search will be updated continuously until completion of the final report. Second, to identify experimental or observational studies on exposure and anxiety, we will employ an additional search strategy based on reference list searches of relevant literature and knowledge of experts in the field of anxiety disorders (contact with experts will be documented). The reasoning for this additional search is as follows: While clinical trials on anxiety among youth are typically conducted within the last one or two decades, observational studies on exposure tend to be somewhat outdated, which is associated with poor indexing in databases or different indexing standards (indexing bias). Therefore, an additional non-systematic search strategy will be employed to identify laboratory or field studies on exposure and anxiety. In addition, reference lists of all identified articles will be hand-searched for potentially relevant studies (“backward citation search”) and all articles that cite any of the identified articles will be retrieved from the Social Science Citation Index (“forward citation search”).

### Data management

EndNote X8 (Thomson Reuters, New York, NY, USA) will be used to manage references and to identify and delete duplicates. All results of the abstract and full-text review including information on the reasons for exclusion during full-text review will be recorded.

### Selection of studies

Selection of studies will be conducted by three of the authors (KSc, KSo, ML). First, titles and abstracts of all retrieved articles will be screened based on the previously defined inclusion and exclusion criteria and assessed for eligibility. Irrelevant articles will be excluded. Next, full-texts of potentially relevant articles will be assessed for eligibility. Reasons for exclusion will be recorded. If full-text articles will be unavailable, first and corresponding authors will be contacted to obtain full-text articles. Authors will be contacted by email (maximum of three attempts). Contact attempts will be recorded. Articles will be distributed in a way that each article will be independently assessed by at least two authors.

### Data extraction

Data extraction will be conducted by three of the authors (KSc, KSo, ML). Data will be extracted from eligible studies independently by at least two authors using a standardized digital data extraction form, which will be piloted and revised during the preliminary search phase. Different sections of the form will document study details including information regarding study identification (e.g., first author, year of publication), study characteristics (e.g., study aims, study design, country in which data were collected), comparators (e.g., conditions), sample characteristics (e.g., age, gender ratio, sample size, methods of recruitment, attrition), patient information, (e.g., diagnoses, comorbidity, diagnostic instruments used), and treatment characteristics (e.g., setting, generic vs. disorder-specific treatment, treatment delivery, use of treatment manual). As parameters of exposure, we will extract whether exposure with “a fear eliciting object” (in vivo or in sensu) has been conducted within or outside of the treatment session according to the treatment manual. We will collect the number of exposure sessions (within-treatment exposure and homework assignment) as well as the length of exposure (in minutes, based on the treatment manual). As outcome characteristics, we will collect the specific outcome measures and the measurement time points. As statistical parameters, we will collect recovery rates, means, standard deviations, and sample size for each group. The application of the data extraction form will be trained beforehand. Disagreements at any stage of this process will be resolved through discussion with the other authors. If disagreements cannot be solved, a fourth reviewer (SS) will serve as an arbitrator. In cases of missing data or unclear information, authors will be contacted for additional information. Again, authors will be contacted by email (maximum of three attempts) and contact attempts will be recorded.

### Risk of bias assessment

The methodological quality of included studies will be measured through a risk of bias assessment conducted by three of the authors (KSc, KSo, ML). For RCTs, the Risk of Bias Assessment Tool (Rob2.0), developed by the Cochrane Collaboration, will be used to assess the risk of bias across six domains including sequence generation, allocation concealment, blinding, incomplete data, selective reporting outcomes, and other sources that may increase the potential risk of bias [30]. For non-randomized studies, the Newcastle-Ottawa Scale (NOS) tool will be used to assess the risk of bias over three domains [31].

### Outcomes

We are primarily interested in outcomes that measure change in anxiety after exposure (e.g., from pre- to post-/follow-up measurement), typically defined as recovery (dichotomous variable) or change in anxiety levels (continuous variable). The effect measures of choice will be odd ratios for dichotomous data and standardized mean differences (SMD) for continuous data for controlled intervention studies. We will collect the primary outcome variable as defined by the respective study. If no primary outcome variable is defined by the study, we will extract up to three anxiety-specific outcomes based on the most commonly used rating scales across studies in this review. If several outcomes are extracted, the average effect size will be calculated. If no anxiety-specific outcome will be reported, we will collect up to three general outcome measures using the same approach. Again, if several outcomes are extracted, the average effect size will be calculated. Outcomes will be collected for all comparators for both intent-to-treat and completer-analyses. No restrictions will be set with regard to type of outcome, type of measurement, or timing of measurement.

### Data synthesis

First, structured narratives and/or summary tables will be used for qualitative/narrative synthesis of studies (e.g., to summarize study characteristics and explore heterogeneity descriptively). All data will be quantitatively synthesized using the freely available statistical software R. A random-effects meta-analysis model will be considered for combining the estimated effect sizes. Meta-regression will be employed to yield a regression coefficient indicating the strength of the association between the degree of exposure and the intervention effect. In addition, we plan to conduct subgroup analyses for age of participants (child samples: on average 12 years or below vs. adolescent samples: on average 13 years or above) to examine whether the role of exposure in treatment efficacy will be greater in younger compared to older children. If insufficient data is available to calculate an effect size, we will include the study for descriptive purposes only and omit the study from the meta-analysis.

### Additional analyses

In addition, we will conduct a sensitivity analysis to evaluate the results of only high-quality studies based on the risk-of-bias assessment. The sensitivity analysis will involve comparison of separately run meta-analyses (e.g., one meta-analysis with all eligible studies and one excluding studies that may confound the meta-analysis such as studies with a high risk of bias or a small sample size).

### Meta-bias(es)

Publication bias will be addressed by visual inspection of funnel plots and by Egger’s test [32]. Statistical heterogeneity in study results will not be quantified, as we expect that study effects will differ as a function of the degree of exposure.

## Discussion

The proposed systematic review and meta-analysis aims to examine the role of exposure in the efficacy of cognitive behavioral interventions for childhood ADs. Any amendments with regard to this protocol when conducting the analyses will be outlined and reported in the final manuscript. As a large body of evidence supports CBT as an effective treatment for ADs in children and adolescents, treatment research should move towards investigating the mechanisms underlying CBT (i.e., *how* treatment works instead of *if* treatment works). With regard to treatment mechanisms, it is still unclear which of the cognitive and behavioural elements of CBT contribute to treatment efficacy. Answering this question will have important clinical implications with regard to improving treatment effectiveness. Scientific information about the role of exposure in treatment success might help practitioners to decide whether to include exposure or not. Moreover, it might help practitioners to plan exposure treatments in more detail and to make evidence-based decisions regarding the frequency, intensity, and qualitative aspects of exposure.

The proposed review may have several limitations at study level as well as at review level. At study level, the majority of relevant clinical trials have been designed with the purpose of testing the efficacy of CBT, with exposure not being the primary focus of interest. Therefore, we expect individual studies to be heterogeneous with regard to how exposure was conducted. The reconstruction of the parameters of exposure might prove difficult, particularly in trials where therapy is delivered under conditions of clinical routine (i.e., with more variation in the delivery of exposure). Moreover, parameters of exposure might not be well-described in publications because CBT is usually delivered as a package of elements with exposure being only one ingredient. As no prior review has attempted a detailed coding of exposure-related parameters, there is no prior experience regarding the availability of data. At review level, the statistical combination of the results of heterogeneous studies into a summary effect may be questionable. In case of evidence of large heterogeneity between studies, interpretation of estimates may be misleading.

A major strength of the proposed review lies in its novelty. This systematic review and analysis will provide empirical evidence on the efficacy of exposure in treating ADs and reducing anxiety symptoms among children and adolescents. In accordance with the scientist-practitioner approach, we hope that our results will help practitioners to make evidence-based decisions regarding the delivery of exposure-based treatments in ADs among youth.

## Supplementary information

**Additional file 1:.** PRISMA 2009 Checklist

**Additional file 2:.** Pubmed search string

## Data Availability

Not applicable.

## Abbreviations

AD: Anxiety disorder
CBT: Cognitive behavior therapy
