# Supplementary material for: The role of exposure in the treatment of anxiety in children and adolescents: protocol of a systematic review and meta-analysis
Source: Syst Rev. 2020 Apr 27;9:96. doi: 10.1186/s13643-020-01337-2 (PMC7187487; doi:10.1186/s13643-020-01337-2)
Supplement: Supplementary file 2 — Additional file 2:. Pubmed search string [file 13643_2020_1337_MOESM2_ESM.docx]

**Additional file 2: Pubmed search string**

((((((((((((child[MeSH Terms])

OR child*[Title/Abstract])

OR adolescent[MeSH Terms])

OR adolescen*[Title/Abstract])

OR youth*[Title/Abstract])

OR young[Title/Abstract])

OR pediatrics[MeSH Terms])

OR pediatric*[Title/Abstract])

OR paediatric*[Title/Abstract])

OR teen*[Title/Abstract])

OR kid*[Title/Abstract])

OR juvenile[Title/Abstract])

AND

(((((((((((((((((((((((((anxiety[MeSH Terms])

OR anxiety[Title/Abstract])

OR anxious[Title/Abstract])

OR "anxiety disorders"[MeSH Terms])

OR "anxiety disorder*"[Title/Abstract])

OR "specific phobia"[Title/Abstract])

OR "simple phobia"[Title/Abstract])

OR "phobia, social"[MeSH Terms])

OR "social phobia"[Title/Abstract])

OR "social anxiety"[Title/Abstract])

OR "anxiety, separation"[MeSH Terms])

OR "separation anxiety disorder*"[Title/Abstract])

OR "generalized anxiety disorder*"[Title/Abstract])

OR "overanxious disorder*"[Title/Abstract])

OR "avoidant disorder*"[Title/Abstract]) OR "mutism"[MeSH Terms])

OR "selective mutism"[Title/Abstract])

OR "elective mutism"[Title/Abstract])

OR "panic disorder"[MeSH Terms])

OR "panic disorder*"[Title/Abstract])

OR "agoraphobia"[MeSH Terms])

OR agoraphobia[Title/Abstract])

OR "phobic disorders"[MeSH Terms])

OR "phobic disorder*"[Title/Abstract])

OR "school refusal"[Title/Abstract])

OR "school avoidance"[Title/Abstract])

AND

((((((((("psychotherapy"[MeSH Terms])

OR psychotherapy[Title/Abstract])

OR therapy[Title/Abstract])

OR intervention*[Title/Abstract])

OR treatment*[Title/Abstract])

OR CBT[Title/Abstract])

OR training*[Title/Abstract])

OR exposure [Title/Abstract])

OR confrontation [Title/Abstract])

AND

((((((((((((((((((((((((((((("randomized controlled trials as topic"[MeSH Terms])

OR "controlled clinical trials as topic"[MeSH Terms])

OR "randomized controlled trial*"[Title/Abstract])

OR "randomised controlled trial*"[Title/Abstract])

OR "randomized-controlled trial*"[Title/Abstract])

OR "randomised-controlled trial*"[Title/Abstract])

OR "randomized control trial*"[Title/Abstract])

OR "randomised control trial*"[Title/Abstract])

OR "randomized-control trial*"[Title/Abstract])

OR "randomised-control trial*"[Title/Abstract])

OR RCT [Title/Abstract])

OR "randomized trial*"[Title/Abstract])

OR "randomised trial*"[Title/Abstract])

OR "quasi randomized trial*"[Title/Abstract])

OR "quasi-randomized trial*"[Title/Abstract])

OR "quasi randomised trial*"[Title/Abstract])

OR "quasi-randomised trial*"[Title/Abstract])

OR "cluster randomized trial*"[Title/Abstract])

OR "cluster-randomized trial*"[Title/Abstract])

OR "cluster randomised trial*"[Title/Abstract])

OR "cluster-randomised trial*"[Title/Abstract])

OR "cluster trial*"[Title/Abstract])

OR "cluster-trial*"[Title/Abstract])

OR "cluster randomized stud*"[Title/Abstract])

OR "cluster-randomized stud*"[Title/Abstract])

OR "cluster randomised stud*"[Title/Abstract])

OR "cluster-randomised stud*"[Title/Abstract])

OR “controlled clinical trial”[Title/Abstract])
